# Sausage Preservation Using Films Composed of Chitosan and a Pickering Emulsion of Essential Oils Stabilized with Waste-Jujube-Kernel-Derived Cellulose Nanocrystals

**DOI:** 10.3390/foods13213487

**Published:** 2024-10-30

**Authors:** Haoyu Chen, Keqi Xin, Qunli Yu

**Affiliations:** College of Food Science and Engineering, Gansu Agricultural University, Lanzhou 730070, China

**Keywords:** bio-composite films, Pickering emulsion, essential oils, sausage preservation

## Abstract

The purpose of this study was to prepare Pickering emulsions stabilized by waste jujube kernel cellulose nanocrystals (CNC) using composite essential oils (EOs) (i.e., cinnamon essential oil [CIN] combined with clove essential oil [CL]). The Pickering emulsions were blended with chitosan (CS) to generate a composite film (CS/CNC/EOs Pickering emulsions). We evaluated the mechanical properties, barrier properties, and microstructures of CS/CNC/EOs bio-based packaging films containing different concentrations of EOs. In addition, the fresh-keeping effects of the composite membranes on beef sausages were evaluated over a 12-day storage period. Notably, the EOs exhibited good compatibility with CS. With the increase in the EOs concentration, the droplet size increased, the composite films became thicker, the elongation at break decreased, the tensile strength increased, and the water vapor permeability decreased. When the composite films were used for preserving beef sausages, the antioxidant and antibacterial activity of the membranes improved as the concentration of EOs increased, effectively prolonging the shelf life of the sausages. Composite membranes with an EOs concentration of 2% exerted the best fresh-keeping effects. Overall, owing to their antioxidant and antimicrobial properties, the bio-based composite films prepared using CS/CNC/EOs Pickering emulsions demonstrated immense potential for application in the packaging of meat products.

## 1. Introduction

Challenges such as bacterial contamination, fat oxidation, and protein oxidation lead to the deterioration of meat quality and reduce the shelf life of meat products. Sausages are common meat products enjoyed by consumers in several countries and primarily contain water, proteins, and lipids. However, due to this complex nutritional composition, the sensory and nutritional qualities of sausages tend to deteriorate during processing and storage [1,2]. The addition of antioxidants and preservatives is considered the most effective approach for ensuring the quality of meat products, especially sausages, and extending their shelf life [3]. However, synthetic preservatives and antioxidants—such as BHA, BHT, nitrates, and nitrites—carry potential risks, including DNA damage, mutations, carcinogenicity, allergic reactions, and nervous system damage [4,5]. Additionally, cooking meat at high temperatures can cause some of these substances (e.g., nitrites) to produce carcinogens [6]. Therefore, the prolonged or excessive consumption of these synthetic additives can negatively impact human health. As a result, natural alternatives, such as plant extracts, are becoming increasingly important for enhancing food safety and protect consumer health [7].

Essential oils (EOs), as secondary plant metabolites, are recognized as natural bioactive compounds due to their powerful antioxidant and antimicrobial properties. Furthermore, they are classified as “Generally Recognized as Safe (GRAS)” by the U.S. FDA due to their high biocompatibility [8]. Cinnamon essential oil (CIN) is primarily composed of cinnamaldehyde, which demonstrates antimicrobial activity against both bacteria and fungi and is thus a promising natural food preservative [9]. Meanwhile, clove essential oil (CL)—a fragrant and volatile oil obtained from the buds of clove flowers—contains a high concentration of eugenol, a compound known for its antimicrobial, antioxidant, and anti-inflammatory properties [10]. Zhao et al. [11] developed potato starch- and polyvinyl-alcohol-based membranes incorporating a CL Pickering emulsion. They observed that these film systems improved the stability of CL and exhibited strong antimicrobial properties. However, the use of individual EOs is associated with some limitations, such as low odor acceptance and restrictions on usage concentrations [12]. Therefore, the combined application of different EOs has garnered widespread attention [13].

Disposable petroleum-based plastics are commonly used for preserving and packaging meat products due to their convenience and low production costs. However, these plastics have several drawbacks, including poor recyclability, being environmentally pollutive, and posing a risk of microplastic contamination [14]. To address these challenges, films based on various biopolymers, including chitosan (CS), cellulose derivatives, starch, and pectin, have been developed [15,16]. These bio-based films can serve as carriers for biodegradable and bioactive compounds, extending the shelf life of food items and preventing environmental pollution. CS, a positively charged polysaccharide derived from chitin through alkaline N-deacetylation, is commonly used in the preparation of thin films due to its excellent film-forming abilities, antimicrobial properties, and biocompatibility [17]. To enhance the functionality of CS-based films, natural antimicrobials and metal nanoparticles have been incorporated into the CS matrix [18,19]. In particular, plant EOs have proven effective in improving both antimicrobial and antioxidant activities and water resistance in bio-based films. In turn, the incorporation of plant EOs into bio-based films can reduce their volatility [20]. However, EOs are often insoluble in the matrices of hydrophilic or acidic membranes, can aggregate on the membrane’s surface, and can be deactivated during membrane drying and storage. These challenges limit the applications of EOs in bio-based food-packaging membranes. However, to solve this problem, EOs can be formulated into oil-in-water (O/W) Pickering emulsions. This approach improves the adhesion of EOs within the film matrix and regulates their release rate [21]. Unlike conventional emulsions, Pickering emulsions use solid particles as stabilizers to enhance the stability of the O/W mixture. These solid particles settle at the interface between the oil and water phases, stabilizing oil droplets through electrostatic and steric mechanisms [22]. Pickering emulsions also offer long-term stability and prevent agglomeration, enhancing the overall functionality of bio-based films [23]. Colloidal particles from various polymers, including natural proteins and polysaccharides, can be used to generate Pickering emulsions. Among these, cellulose nanocrystals (CNC) are particularly effective as stabilizers [24,25].

CNC can be extracted from lignocellulosic materials, bacteria, and algae. They demonstrate very high mechanical strength, biocompatibility, and reliable reproducibility as particle emulsifiers [26]. Jujube is an important economic crop in China, and about 200,000 tons of jujube kernels are produced in the country annually. However, only a fraction of these jujube kernels are used in traditional Chinese medicine, and most of the fiber-rich kernels are discarded during industrial processing [27,28]. Extracting CNC from waste jujube kernels can improve the overall economic value of jujube trees and reduce resource wastage. Studies have demonstrated that jujube-kernel-derived CNC have advantages such as high aspect ratios, large surface areas, and strong hydrophilicity [29,30]. In addition, the incorporation of CNC into CS films can enhance the latter’s mechanical properties. CNC can be uniformly dispersed in the CS matrix at the nanoscale level and form hydrogen bonds with the molecular chains of CS. CNC can thus promote load transfer and stress distribution in CS films, enhancing the cohesion and stability of the film network [25,31,32].

In this study, EOs nanoemulsions with different contents of CIN and CL were prepared to fabricate CS/CNC/EOs Pickering emulsion composite films, with CS serving as the wall material. The physical properties and structural characteristics of each composite film were analyzed, and their effectiveness in preserving sausages was subsequently assessed. The findings of this study highlight the value of bio-based antimicrobial and antioxidant composite films as innovative green materials for packaging meat and meat products. Further, they offer a sustainable and cost-effective solution for reducing environmental pollution and extending the shelf life of food products.

## 2. Materials and Methods

### 2.1. Materials

Cinnamon essential oil (CIN) (Cinnamaldehyde ≥ 85.0%) and clove essential oil (CL) (Eugenol ≥ 85.0%) were purchased from Sinopharm Chemical Reagent Co., Ltd. (Shanghai, China). Chitosan (CS) (deacetylation degree ≥ 95%), glacial acetic acid (CH_3_COOH, ≥ 99.5%), and glycerol (≥99.5% purity) were provided by Macklin (Shanghai, China) Biochemical Science and Technology Co., Ltd. Waste jujube pits remaining after the production of crisp jujubes at Chengdu Shuangliu Zidong Dried Fruit Company were procured for this study. All the other chemicals used in this study were of analytical grade. *Bacillus cereus* (ATCC 14579), *Salmonella enterica* (ATCC 12002), *Staphylococcus aureus* (ATCC 6538), *Escherichia coli* (ATCC 8739), *Aspergillus niger* (ATCC 16404), and *Pseudomonas aeruginosa* (ATCC 9027) were obtained from Gansu Agricultural University (Gansu, China). Fresh beef and pork fat were purchased from nearby supermarkets, and the samples were transported to our laboratory in a polystyrene cooling box within 45 min.

### 2.2. Minimum Inhibitory Concentration (MIC) of the Essential Oils

The minimum inhibitory concentration (MIC) of the EOs was determined using the two-fold dilution method described by Wiegand et al. [33], with slight modifications. Under aseptic conditions, EOs (1.00 mL), diluted with acetone, were added to a petri dish containing 19 mL of medium. The volume of acetone was adjusted to achieve final EOs concentrations of 2, 1, 0.5, 0.25, 0.125, 0.0625, and 0.0313 μL/mL. Meanwhile, 1 mL of acetone—without any EOs—was added to the control group. The Petri dishes were labeled, and 100 μL of the appropriate microbial suspension was added to the plate and spread evenly on the surface of the medium. Plates containing bacterial cultures were incubated at 37 °C for 24 h, whereas those containing fungal cultures were incubated at 28 °C for 48 h. Microbial growth was observed, and the lowest concentration of EOs that inhibited microbial growth was designated as the MIC. All the experiments were repeated thrice.

### 2.3. Synergistic Effect of the Essential Oils

The checkerboard method was chosen to assess the possible synergistic effects of CIN and CL [12]. The MIC values of CIN and CL for the different bacteria to be tested were determined separately, and the essential oils for the combined assay were diluted based on the MIC values of CIN and CL. Sixteen sterile test tubes were labeled and arranged in a 4 × 4 grid. Then, 5 mL of liquid culture medium was added to all tubes. CIN (vertical axis serially diluted two-fold, horizontal axis unchanged) was added along the vertical axis, while CL (horizontal axis serially diluted two-fold, vertical axis unchanged) was added along the horizontal axis. The final diluted concentrations of the EOs were 1/8 MIC, 1/4 MIC, 1/2 MIC, and MIC, respectively. Then, 100 μL of the appropriate microbial suspension was added to each tube. Tubes containing bacterial cultures were incubated at 37 °C for 24 h, while those containing fungal cultures were incubated at 28 °C for 48 h. All measurements were obtained in duplicate and repeated, and mean values were obtained accordingly. The fractional inhibitory concentration (FIC) index (FICI) was calculated as follows:


FIC Index = FIC (A) + FIC (B)

$$\mathrm{FIC}\;(A)=\frac{MIC(A)\;in\;combination}{MIC(A)\;alone}$$

$$\mathrm{FIC}\;(B)=\frac{MIC(B)\;in\;combination}{MIC(B)\;alone}$$

Based on the FICI, we demonstrated that the EOs showed synergism (FICI ≤ 0.5); additive effects (FICI = 0.5–1.0); indifference (non-interactive effects) (FICI = 1.0–4.0); or antagonism (FICI > 4.0).

### 2.4. Bacteriostatic Effects of the Essential Oils

The antibacterial activity of the EOs was analyzed using the disk diffusion method [34]. A sterile filter paper disk (diameter = 1 cm) was prepared. Drops of pure EOs, either CIN or CL or a combination thereof (CIN:CL = 1:1; CIN:CL = 1:2; CIN:CL = 1:3; CIN:CL = 2:1; CIN:CL = 3:1, *v*/*v*), were added to the filter paper. This filter paper was then placed on a beef extract peptone agar plate previously coated with 0.1 mL of a 10^6^ CFU/mL bacterial culture derived from spoilt sausages. The plates were incubated at 37.0 ± 0.1 °C for 24 h. Subsequently, the diameter of the inhibition zone was measured to evaluate the antibacterial activity of the EOs against the bacterial strains. This experiment was repeated thrice.

### 2.5. Antioxidant Capacity of the Essential Oils

The antioxidant capacity of pure EOs (CIN or CL) and composite EOs mixtures (CIN:CL = 1:1; CIN:CL = 1:2; CIN:CL = 1:3; CIN:CL = 2:1; CIN:CL = 3:1, *v*/*v*) was tested using the DPPH method described by Erfanifar Z et al. [35]. In this assay, methanol was used as the blank, and 0.1 mM DPPH in methanol was used as the control. The absorbance of each DPPH-and-EOs mixture was evaluated at a wavelength of 517 nm to determine the antioxidant activity of the EOs.

$$DPPH\;Scavenging\;activity(\%)=\frac{Ac-As}{Ac}\times 100\%$$

Here, Ac and As represent the absorbance values of the control (0.1 mM DPPH in methanol) and sample, respectively.

### 2.6. Preparation of Pickering Emulsions Loaded with Essential Oils

#### 2.6.1. Preparation of Cellulose Nanocrystals

The method described by Wang et al. [29] was used for the preparation of CNC. Jujube kernels were dried and pulverized into a fine powder using a food processor. To obtain CNC, the jujube kernels were subjected to various treatments. First, the jujube kernel powder was subjected to Soxhlet extraction with a mixture of ethanol and benzene (1:2 ratio) at 85 °C for 8 h (with reflux) for dewaxing. The dewaxed jujube kernels were shaken in 10% sodium hydroxide for 4 h at 90 °C in a water bath, filtered, and washed and dried to obtain delignified jujube kernels. They were then bleached with glacial acetic acid for 1 h at 80 °C in a water bath and shaken. The mixture was cooled to room temperature, filtered through No. 42 Whatman filter paper, and washed with distilled water until the filtrate became neutral. The bleached jujube kernels were dried at 45 °C for 24 h and then hydrolyzed with 63.5% sulfuric acid for 40 min before being washed and dried to obtain jujube kernel CNC.

#### 2.6.2. Preparation of Pickering Emulsions

Pickering emulsions were formulated based on the technique developed by Guo et al. [36], with a minor alteration. The CNC suspension was diluted to a concentration of 1.0% (wt.%) using ultra-pure water and treated with ultrasound for 10 min. CIN and CL were combined in different proportions (pure CIN; pure CL; CIN:CL = 1:1; CIN:CL = 1:2; CIN:CL = 1:3; CIN:CL = 2:1; CIN:CL = 3:1, *v*/*v*), and the antimicrobial and antioxidant activities of these combinations were tested to identify the most suitable EO composition. The EOs were added to a 1.0% (wt.%) CNC suspension at concentration ratios of 5%, 10%, 15%, and 20% (*w*/*w* CNC). The samples were emulsified using a high-shear homogenizing emulsifier (FJ200-SH, Shanghai Huxi Industry Co., Shanghai, China) at 12,000 rpm for 5 min to obtain a crude emulsion. The samples were then ultrasonicated for 10 min to obtain the final emulsion, which was kept at 4 °C before subsequent use. The final Pickering emulsions were designated as CNC, CNC/EOs-5, CNC/EOs-10, CNC/EOs-15, and CNC/EOs-20 according to their EOs concentrations.

### 2.7. Characterization of Pickering Emulsions—Particle Size, Polydispersity Index (PDI), ζ-Potential, and Morphology

The Zetasizer Nano ZS system (Malvern Instruments Inc., Worcestershire, UK) was used to analyze the PDI and ζ-potential of the Pickering emulsions. Prior to measurement, the samples were diluted 100-fold using ultrapure water in order to mitigate multiple scattering effects. The emulsions were added to a glass slide and observed using a light microscope (JEM-1200EX, Hitachi, Tokyo, Japan). The size of the particles was determined by analyzing microscopy images using the Nano Measurer 1.2 software.

### 2.8. Preparation of CS/CNC/EOs Pickering Emulsion Composite Films

**Figure 1:**
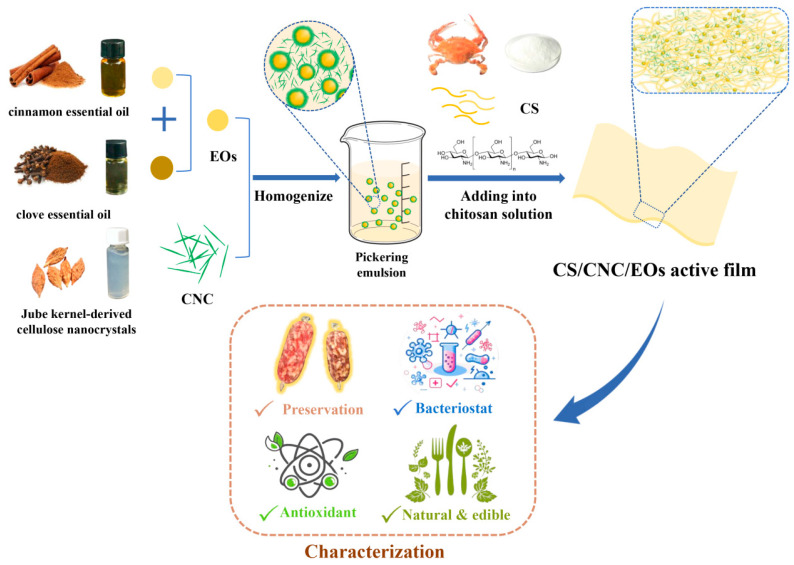
Schematic showing the synthesis of fully bio-based antimicrobial and antioxidant membranes using chitosan combined with a Pickering emulsion of essential oils stabilized with cellulose nanocrystals.

The CS/CNC/EOs Pickering emulsion composite films were prepared by slightly modifying the method employed by Rui et al. [37]. The schematic diagram in Figure 1 illustrates the preparation of the fully bio-based active films containing the EOs Pickering emulsions. First, 1.4 g of CS was dissolved in 100 mL of glacial acetic acid (0.8% *v*/*v*) and stirred with a magnetic agitator (DF-101S) at room temperature for 30 min to prepare the CS solution (1.4% *w*/*v*). Then, 5% glycerin (*w*/*w* CS) was added to this solution, and the mixture was stirred for 30 min. Subsequently, the Pickering emulsions containing different concentrations of EOs (prepared as described in Section 2.6.2) were added to the CS solution to obtain composite membranes with final EOs concentrations of 0, 0.5, 1.0, 1.5, and 2% (*v*/*v*) in CS. The film-forming solution was agitated using a magnetic stirrer to ensure homogeneity, and the mixture was sonicated to remove any bubbles. Each film-forming solution was poured into a mold and placed in an oven (DHG-940A) at 40 °C for 24 h. The final films were labeled as CS/CNC, CS/CNC/EOs-5, CS/CNC/EOs-10, CS/CNC/EOs-15, and CS/CNC/EOs-20. A film lacking EOs was used as the control. The films were cut into squares using a knife, and the outer edges of the film sheets were sealed with the corresponding film solutions. The sheets were dried at room temperature for 3 days to ensure complete solvent evaporation, and tubular casings were prepared for sausage filling (diameter × length: 3 cm × 15 cm). Prior to further assessment, all films were subjected to 48 h of conditioning at a temperature of 25 °C and relative humidity of 50%.

### 2.9. Characterization of CS/CNC/EOs Pickering Emulsion Composite Films

#### 2.9.1. Film Thickness and Mechanical Properties

The thicknesses of the films were examined at ten random positions using a handheld digital thickness gauge (Nscing-4512, Nanjing Nscing Measuring Instrument Co., Nanjing, China) with an accuracy of 0.001 mm. The tensile strength (TS) and elongation at break (EAB) were measured with a texture analyzer (TA. XT Plus, Isenso, Atlanta, GA, USA) based on the method described by Cao et al. [38]. First, the films were cut into 60 mm × 10 mm rectangles. During testing, the initial distance between the two fixtures was 20 mm, and the stretching speed was 10 mm/min.

#### 2.9.2. Water Vapor Permeability (WVP)

The WVP of the membranes was assessed using the method proposed by Fan et al. [39]. For this experiment, 10 g of anhydrous calcium chloride was added to a beaker (diameter = 55 mm), and the beaker was sealed with the CS/CNC/EOs Pickering emulsion composite film. The beaker was then stored at 25° C and 75% relative humidity under ambient conditions. The beaker was checked every 12 h for 2 days to determine the loss of mass. WVP was calculated according to the following formula:

$$\mathrm{WVP}\;(g/(m\;s\;\mathrm{Pa}))=\frac{\Delta S\times d}{t\times A\times \Delta P}$$

Here, ΔS is the mass loss (g), d is the film thickness (m), t is the measurement time point (s), A is the mouth area of the beaker (mm^2^), and ΔP is the pressure difference between the inside and outside of the film-sealed beaker (3167.1 Pa at 25 °C).

#### 2.9.3. Color Properties

The color properties of the films were determined using a CM2300D colorimeter (Konica Minolta Investment Co., Ltd., Tokyo, Japan) according to the Chinese standard GB/T 7921-2008 [11]. The CIE Lab standards, which include L* (lightness), a* (redness/greenness), and b* (yellowness/blueness), were used to assess the color of the films. The color deviation (ΔE) from the white reference was evaluated using the following equation:

$$\Delta E=\sqrt{(L^{*}-{L_{0})}^{2}+(a^{*}-{a_{0})}^{2}+(b^{*}-{b_{0})}^{2}}$$

Here, *L*_0_ = 97.84, *a*_0_ = −0.23, and *b*_0_ = 0.31 are the color parameters of the calibration plate, and L*, a*, and b* are the color parameters of the films.

#### 2.9.4. Scanning Electron Microscopy (SEM)

SEM is a technique used to examine the surface morphologies of objects at a high resolution. A JSM-IT700HR scanning electron microscope (JEOL, Tokyo, Japan) was used to observe the surfaces of the composite films at a magnification of 500×. The cross-sections of the films were subsequently observed at a magnification of 2000×.

#### 2.9.5. Fourier-Transform Infrared Spectroscopy (FTIR)

The infrared spectra of the samples were measured using an FTIR spectrometer (Spectrum 400, Perkin Elmer, Waltham, MA, USA). The films were scanned at a resolution of 4 cm^−1^, and absorption spectra in the range of 4000–400 cm^−1^ were recorded.

#### 2.9.6. Thermal Analysis

The films were examined using differential scanning calorimetry (DSC) (DSC-3, Mettler Toledo, Greifensee, Switzerland). Film samples (5 mg) were placed on an aluminum tray and heated from 20 °C to 180 °C at a heating rate of 10 °C/min under a nitrogen flow rate of 30 cm^3^/min [40].

### 2.10. Analysis of the Fresh-Keeping Effects of the Composite Membranes

#### 2.10.1. Sausage Preparation

The workbench and all instruments were sterilized using 75% ethanol. The sausages were composed of 70% lean beef, 28% pork fat, and 2% salt; the beef and fat were minced and mixed thoroughly, and the sausage stuffing was filled into the finished film tubes. The ends of the sausages were tightly secured with sterile cotton threads. The average length of the sausages was 8.2 ± 0.27 cm. All samples were stored at 4 ± 1 °C and analyzed after 0, 3, 6, 9, and 12 days of storage. The sausage samples were analyzed immediately after tearing off the CS/CNC/EOs films. All measurements were repeated using three different samples.

#### 2.10.2. pH Value

The pH values of the sausage samples were determined using an automated digital pH meter (PB-10; Sartorius, Berlin, Germany) and verified using standard buffer solutions (pH = 4.00 and 7.00) at a temperature of 20 °C. Specifically, 5 g of each sample was thoroughly mixed with 25 mL of distilled water for 30 s, and the pH value was measured thereafter [41].

#### 2.10.3. Thiobarbituric Acid Reactive Substances (TBARS) Value

The TBARS value was evaluated based on the methodology described by Cao et al. [41]. A spectrophotometer (UV-756P, Shimadzu, Kyoto, Japan) was used to measure the TBARS value at a wavelength of 532 nm. The results were expressed as milligrams of malondialdehyde (MDA) per kilogram of sample.

#### 2.10.4. Total Volatile Basic Nitrogen (TVB-N) Value

The TVB-N content of the sausages during storage was determined using the method described by Ran et al. [42]. Sausage samples (10 g) were combined with 100 mL of distilled water and vigorously mixed for 30 min. Subsequently, a solution containing 5 mL of MgO (10 g/L) and 10 mL of boric acid was added to the samples along with 2 mL of a mixed indicator (1 g/L of methyl red and 1 g/L of bromocresol green = 1:5). The mixture was then titrated against hydrochloric acid (0.01 M) until the titration endpoint was reached.

#### 2.10.5. Total Viable Bacteria Count (TVC)

The TVC was determined in accordance with the international standards set by AFNOR [43]. First, 10 g of the sausage sample was combined with 90 mL of distilled water, and this mixture was serially diluted. Then, 1 mL of each diluted sample was utilized to determine the TVC using the plate count method. The samples were incubated at a temperature of 37 ± 1 °C for 48 h. The number of colony-forming units (CFU) per gram of sample (CFU/g) was measured.

#### 2.10.6. Sensory Assessment

A trained panel of 17 members performed sensory evaluations of the sausages on days 0, 6, and 12 of cold storage. The evaluations included assessments of appearance, color, odor, texture, and overall acceptability [44]. The sensory assessments were carried out in a dedicated facility with controlled lighting, temperature, and humidity. The sensory assessment was approved by the Institutional Review Board of the College of Food Science and Engineering, Gansu Agricultural University, and written informed consent was obtained from all participants prior to their enrolment in this study.

### 2.11. Statistical Analysis

The data were subjected to analysis of variance (ANOVA) using SPSS version 26. Duncan’s method was employed to conduct post hoc analyses and compare the mean values between different groups. *p* < 0.05 was considered statistically significant.

## 3. Results and Discussion

### 3.1. Synergistic Effects of Cinnamon and Clove Essential Oils

Synergistic effects occur when the antibacterial effect of composite EOs is better than the sum of the effects of individual EOs. Meanwhile, additive effects occur when the antibacterial effect of composite EOs is equal to the sum of the effects of each individual EO. In contrast, irrelevant effects are defined as when the use of composite EOs results in antibacterial effects that are equal to those of the individual EOs. Finally, antagonistic effects are defined as when the antibacterial effect of the composite EOs is weaker than that of one or both individual EOs.

The MIC values of the test oils against different bacterial and fungal strains were examined using the microbroth dilution method. The combined FICI values were determined using the checkerboard titration method. The FICI values (Table 1) demonstrated that the CIN/CL combination had a synergistic antibacterial effect against *Staphylococcus aureus* (FICI: 0.375) and *Bacillus cereus* (FICI: 0.5). Meanwhile, it had additive antibacterial effects against *Escherichia coli* (FICI: 1.0), *Salmonella enterica* (FICI: 0.625), *Aspergillus niger* (FICI: 1.0), and *Pseudomonas aeruginosa* (FICI: 0.75). Encouragingly, the composite EO mixture did not show irrelevant or antagonistic effects against any microbial strain. These findings are consistent with those reported previously [13,45,46]. In conclusion, the EOs containing natural bioactive compounds demonstrated synergistic and additive antimicrobial effects against common food-borne microorganisms. This indicated that they could be incorporated into food-active packaging films as antimicrobial agents.

### 3.2. Optimal Composition of Composite Essential Oil Mixtures

**Figure 2:**
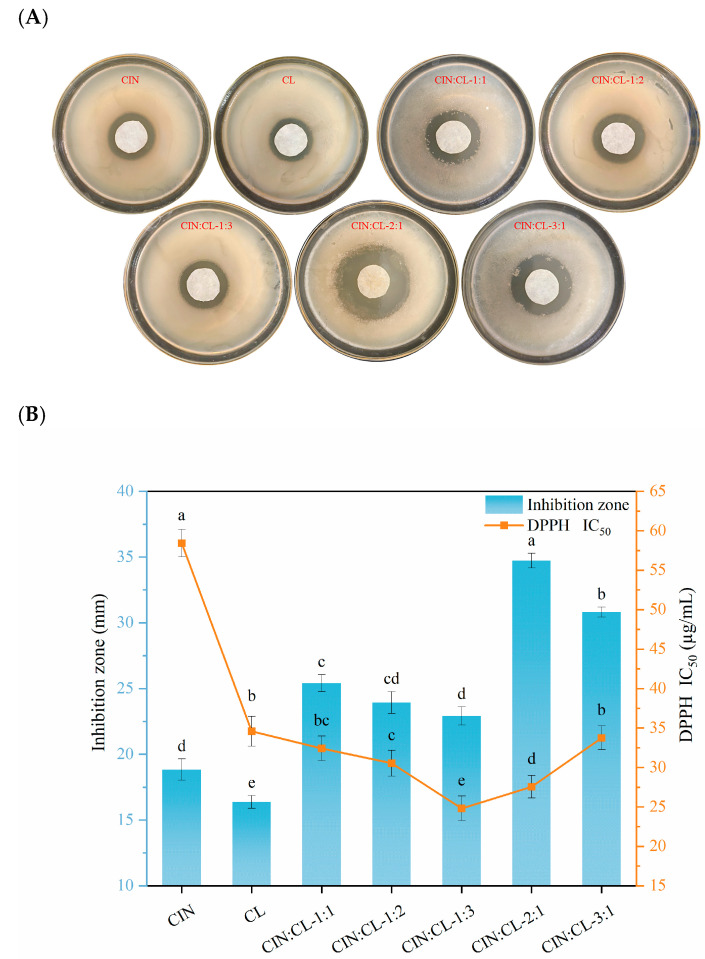
(**A**) Digital photographs showing the antibacterial effect of composites containing different ratios of essential oils. (**B**) Inhibition zone and DPPH IC_50_ value of composites containing different ratios of essential oils. Different letters indicate significant differences (*p* < 0.05).

The synergistic antioxidant effects of CIN and CL were examined to identify the optimal proportion of each EOs in the composite mixture. Figure 2B shows the IC_50_ values of the individual and composite EOs observed in the DPPH radical-scavenging assay. The DPPH radical-scavenging rate of CL was significantly higher than that of CIN, and the IC_50_ values of all the composite EOs were significantly lower (*p* < 0.05) than the average IC_50_ values of their individual components. The DPPH radical-scavenging rate was significantly higher in the CIN:CL = 1:3 and CIN:CL = 2:1 groups than in the pure EO groups and the other composite EOs groups (*p* < 0.05), indicating that these ratios offered better antioxidant effects. These findings are similar to those reported by Purkait S et al. [13], who found that of the various EOs combinations tested, CIN combined with CL provided consistent synergistic antioxidant activity.

The inhibitory effects of the different composite EOs mixtures against the bacteria extracted from the spoiled sausages are illustrated in Figure 2A. As shown in Figure 2B, the diameter of the circle of inhibition was significantly greater after treatment with CIN alone than after treatment with CL alone (*p* < 0.05), corroborating the MIC values shown in Table 1. Sethunga M et al. [47] discovered the synergistic antibacterial effects of the EOs and oleoresins of cinnamon, clove buds, and ginger. In their study, cinnamon peel oil and oleoresins produced the highest zones of inhibition against a range of microorganisms. In the present study, when CIN and CL were combined, the inhibitory effect of the composite EOs improved gradually with the increase in CIN content. However, no linear positive correlation was observed. For example, when the volume ratio between CIN and CL shifted from 3:1 to 2:1, the proportion of CIN decreased, but the diameter of the inhibition zone increased (*p* < 0.05). The best inhibitory effect was achieved when the volume ratio of CIN to CL was 2:1, producing an inhibition zone diameter of 35.13 ± 1.06 mm.

The bioactive compounds responsible for the combined antimicrobial and antioxidant effects of CIN and CL are trans-cinnamaldehyde (also known as (2E)-3-phenylprop-2-enal) and eugenol (also known as 4-allyl-2-methoxyphenol), respectively [13]. Cinnamaldehyde disrupts the integrity of bacterial cell membranes, while eugenol inhibits bacterial enzyme activity and protein synthesis. The synergistic effects of these compounds allow composite EOs to significantly inhibit the growth of foodborne pathogens at lower concentrations than individual EOs [12,13]. Interestingly, Ju et al. [48] concluded that EOs have trace components that contribute toward their antimicrobial effects and produce synergistic effects in combination with the major components. Based on our findings, a 2:1 composite ratio of CIN to CL (CIN:CL = 2:1, V:V) was selected for the final Pickering emulsion. This composite formulation of EOs provided the strongest antimicrobial effects of all formulas and stronger antioxidant activity compared to a single essential oil. Additionally, owing to the lower proportion of CIN in this composite formulation, the odor of cinnamon was reduced and the aroma of clove increased. Further, this formulation minimizes the total use of essential oils for the same antimicrobial effect, which improves the overall acceptability of the composite EOs for application in packaging films for meat products.

### 3.3. Characterization of CNC/EOs Pickering Emulsions

**Figure 3:**
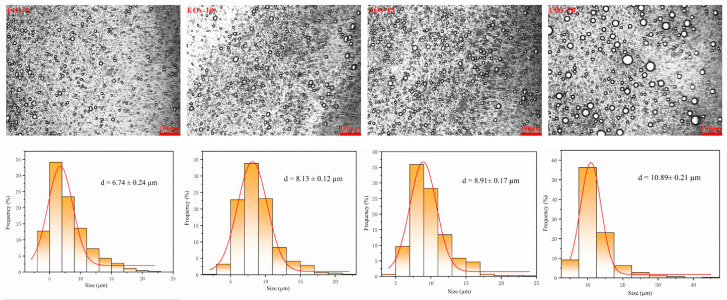
Optical microscopy images and corresponding particle size distributions of Pickering emulsions containing different concentrations of EOs.

The optical microscopy images and particle size distributions of Pickering emulsions prepared using different EOs (CIN:CL = 2:1) concentrations are shown in Figure 3. Notably, the Pickering emulsions contained nearly spherical droplets, and the droplet size was uniform.

**Figure 4:**
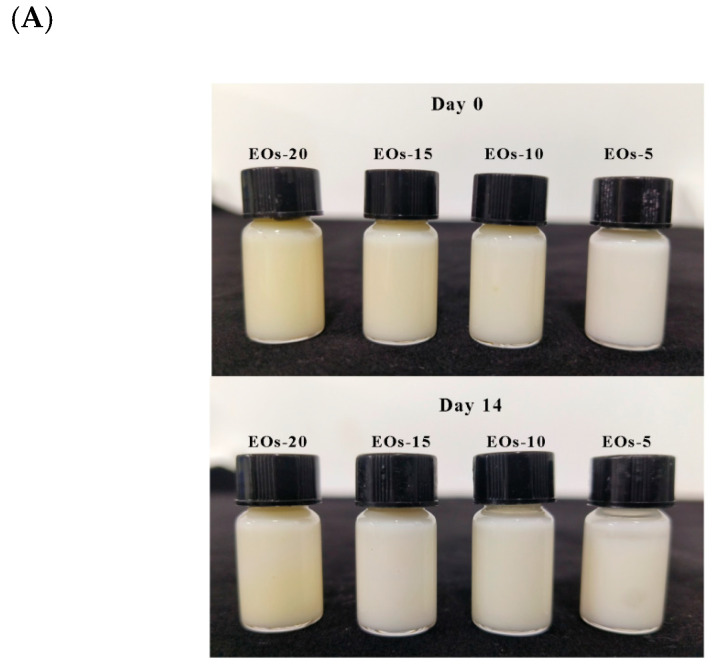

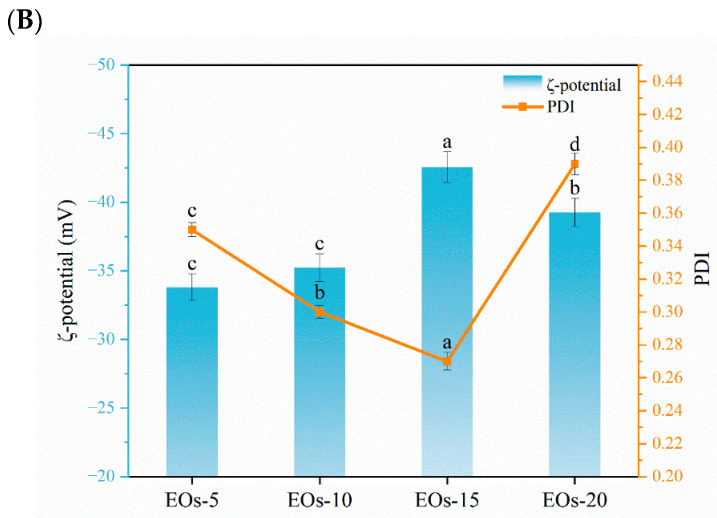
(**A**) Digital photographs of EOs Pickering emulsions after 0 and 14 days; (**B**) PDI and ζ-potential of Pickering emulsions with different concentrations of EOs. Different letters indicate significant differences (*p* < 0.05).

Particle size distribution is an important parameter affecting the stability of Pickering emulsions [49]. In this study, the particle size range of EOs Pickering emulsions was 6.74–10.89 μm. The particle size distribution exhibited a single peak mode, and the observed particle size was consistent with previous reports [39,50]. As the proportion of EOs increased from 5% to 20%, the particle size of the emulsion increased significantly, likely because of stronger binding between the stabilizer (CNC) and the surfaces of the oil droplets [26]. All the prepared Pickering emulsions remained stable and homogeneous, without showing phase separation or droplet merging, throughout 1 month of storage. Nevertheless, the emulsions showed gradual yellowing during this period due to the presence of EOs (Figure 4A).

The PDI reflects the degree of uniformity in the droplet size distribution of emulsions, and PDI values closer to 0 indicate a more uniform size distribution. In this study, the PDI values of the EOs emulsions ranged from 0.26 to 0.39. Additionally, their particle size distribution range was significantly narrower than that of the thyme EOs nanoemulsions prepared by Ghoshal et al. (with a PDI value of 0.305) and the nanofibrous cellulose-stabilized bergamot oil nanoemulsions fabricated by Sogut (with PDI values of 0.53–0.79). This suggested that the EOs emulsions prepared in this study had a uniform particle size distribution [51,52]. Typically, the stability of emulsions improves as the surface charge density increases due to the electrostatic repulsion between the emulsion droplets. Figure 4B shows the ζ-potential of the Pickering emulsions prepared using different concentrations of EOs. Droplets in nanoemulsions are considered stable if their charges are above +30 mV or below −30 mV [37]. The ζ-potentials of the Pickering emulsions prepared with 1.0% CNC-stabilized EOs were all below −30 mV, with the 15% EOs emulsions showing the lowest ζ-potential value (−43.2 mV). The stability and droplet uniformity of the EOs Pickering emulsions were confirmed by their lower negative ζ-potential and PDI values.

After emulsion preparation, we concluded that the CNC extracted from waste jujube kernels provided good physical stability [30] and good coverage, generating a stable Pickering mechanism through spatial site resistance and electrostatic repulsion due to the negative charge of the oil droplets. This negative charge protected the emulsified droplets from agglomerating and enabled the formation of a mechanically strong network around them. Moreover, the presence of hydroxyl and carboxyl groups in the bioactive constituents of the EOs, including eugenol and cinnamic acid, likely enhanced the electrostatic interactions between the droplets. This, in turn, led to improved droplet homogeneity and emulsion stability [53]. Together, the findings show that the EOs Pickering emulsions have good stability and can be used for the preparation of composite films.

### 3.4. Characterization of the Physical Properties of CS/CNC/EOs Pickering Emulsion Films

The thickness of films and coatings is a crucial physical attribute that directly impacts their color, mechanical characteristics, and capacity to prevent the entry of water vapor and other gases [54]. Consequently, this parameter affects the shelf life of stored foods. Table 2 shows the thicknesses, TS, EAB, and WVP of the composite films. The film thickness could be changed by adjusting the solid content after dehydration. Compared with blank films, composite films showed higher thickness. There was a significant difference in thickness (*p* < 0.05) between the blank films (0.114 mm) and the CS/CNC/EOs-20 film (0.161 mm). Moreover, the membrane thickness increased significantly with the increase in EOs concentration (*p* < 0.05), likely due to the addition of EOs nanodroplets to the CS membrane network. The interaction of the EOs Pickering emulsion with the CS matrix may have increased the spatial distance within the chitosan matrix and increased the thickness [55].

The mechanical properties of a film are important because they affect the film’s ability to protect food during transportation, storage, and handling. TS and EAB are two key mechanical indicators reflecting the strength and extensibility of thin films, respectively. These values can be obtained using tensile tests (Table 2). In this study, when the EOs concentrations increased from 0% to 2%, the TS of the films decreased from 19.43 MPa to 15.52 MPa. The TS values of the composite membranes were significantly lower (*p* < 0.05) than those of the blank membranes. Additionally, the EAB values peaked at an EOs concentration of 1.5% (EAB = 20.74%) before decreasing with increasing EOs concentrations thereafter (*p* < 0.05). The reduction in the TS of the films can be attributed to the embedding of the emulsion droplets into the continuous film matrix, which reduced the internal continuity of the film [56]. Weak connections between CS and EOs Pickering emulsions can replace the strong bonds between polymers, increasing the free volume and molecular mobility in the film [57]. This can create fragmented polymer networks, decrease matrix binding, and facilitate membrane breakage, thereby decreasing cohesion and TS. Liu et al. [58] added a cellulose-nanoparticle-stabilized CIN Pickering emulsion to CS films and found that the TS value of the films decreased to 16.42 ± 0.5 MPa (TS value of CS/CNC films = 22.94 ± 0.7 MPa). In addition, the TS value of the cellulose-nanocrystal-stabilized composite film clove bud oil Pickering emulsion developed by S. P. Bangar et al. [22] was found to be 13.43 ± 0.14 MPa, which is close to the TS value of CS/CNC/EOs-20 (15.52 ± 0.59 MPa).

WVP is an important characteristic for evaluating the moisture resistance of food-packaging films. A lower WVP is preferred because it reduces the amount of water packaged foods lose to the atmosphere [59]. The WVP values of the films prepared in this study are shown in Table 2. The WVP value of the composite films containing EOs Pickering emulsions was significantly lower than that of the control films (*p* < 0.05). Specifically, the WVP values of the films with EOs Pickering emulsion concentrations of 0.5%, 1.0%, 1.5%, and 2.0% were 16.67%, 18.72%, 33.11%, and 51.14% lower than that of the control film, respectively. The WVP of a membrane is determined by the hydrophilicity–lipophilicity ratio of the membrane matrix, as water molecules must pass through the hydrophilic portion of the matrix [60]. Therefore, the improved water barrier performance of the membranes containing Pickering emulsions could be related to their hydrophobicity. Notably, the existence of hydrophobic oil droplets can hinder the movement of water vapor across the membrane [58,60]. Furthermore, emulsified EOs are more homogeneously dispersed, increasing the curvature of water molecules and decreasing their ability to cross the membrane, thus reducing the WVP [22].

**Figure 5:**
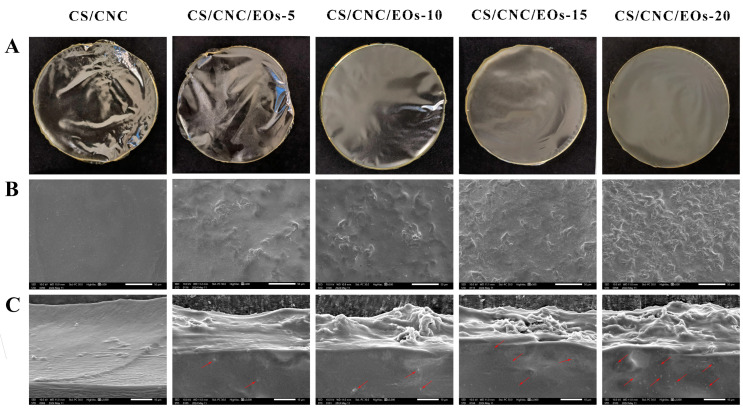
(**A**) Photographs of CS/CNC/EOs films obtained using a digital camera; (**B**) SEM images showing the surfaces of CS/CNC/EOs films; (**C**) SEM images showing the cross-sections of CS/CNC/EOs films.

The color of food-packaging materials has a direct impact on consumers’ purchasing decisions [42]. Table 3 displays the hues of antioxidant and antimicrobial CS/CNC/EOs films. The inclusion of EOs affected various color aspects of the active composite films, including the L*, a*, b*, and ΔE values. As the concentrations of EOs Pickering emulsions in the films increased, the values of a* and b* also increased. Specifically, the a* value increased from −1.46 to 1.23, and the b* value increased from 2.45 to 14.51 (*p* < 0.05). This indicated a shift towards a more yellow color in the films. This yellow color could be attributed to the inherent color of the EOs themselves, as demonstrated by the photographs of the films shown in Figure 5A. The color difference (ΔE) also exhibited a similar trend, escalating linearly with the concentrations of EO. When the EOs concentration was higher, a small increase in both b* and ΔE was noted, consistent with the findings reported by Zhao et al. [11].

### 3.5. Characterization of the Morphologies of CS/CNC/EOs Pickering Emulsion Films

#### 3.5.1. Scanning Electron Microscopy

The microstructural properties of packaging films significantly impact their physical properties, mechanical properties, barrier function, and visual characteristics [61].Figure 5A shows some photographs of the CS/CNC/EOs films obtained using a digital camera. Meanwhile, Figure 5B shows surface and Figure 5C cross-section micrographs of CS films doped with different EOs Pickering emulsions (EOs concentrations of 0%, 0.5%, 1.0%, 1.5%, and 2%). Obvious differences in morphology and surface structure were observed among the different CS films. After the addition of EOs Pickering emulsions, numerous minute particles could be detected in the cross-sections of the films and on their surfaces. As the concentrations of EOs increased, the surfaces of the composite films became increasingly rough due to the presence of more particles and agglomeration. It is possible that the droplets of EOs underwent heightened flocculation and agglomeration during the film-drying process as their content increased, leading to the formation of sizable droplets within the matrix [62]. Subsequently, the drying process likely caused water to evaporate from the films, resulting in the CNC being left behind as the Pickering emulsions migrated to the surface of the polysaccharide network.

Interestingly, the cross-sections of the blank films contained several relatively loose gully structures. However, the films became increasingly dense after the addition of EOs Pickering emulsions. Moreover, the number of EOs particles (red arrow) in the cross-section increased with the increase in the EOs concentration, and the section density became higher. This could be attributed to the filling effect of the EOs Pickering emulsions [36], which increased the compactness of the composite films and affected their mechanical characteristics. The increase in particle concentration could also be due to the heightened emulsification, flocculation, and agglomeration of EOs, as the content of EOs Pickering emulsions increased during the film-drying process, leading to the formation of larger droplets within the matrix. SEM analysis revealed that the blank film had a sleek and consistent outer surface, as well as a compact and uninterrupted internal microstructure, unlike the Pickering emulsion composite films loaded with EOs. These results are consistent with those of prior experiments from this study (TS, EAB, and WVP). Notably, similar outcomes have also been documented for composite films produced using ginger EOs emulsions [63].

#### 3.5.2. Fourier Transform Infrared Spectra

**Figure 6:**
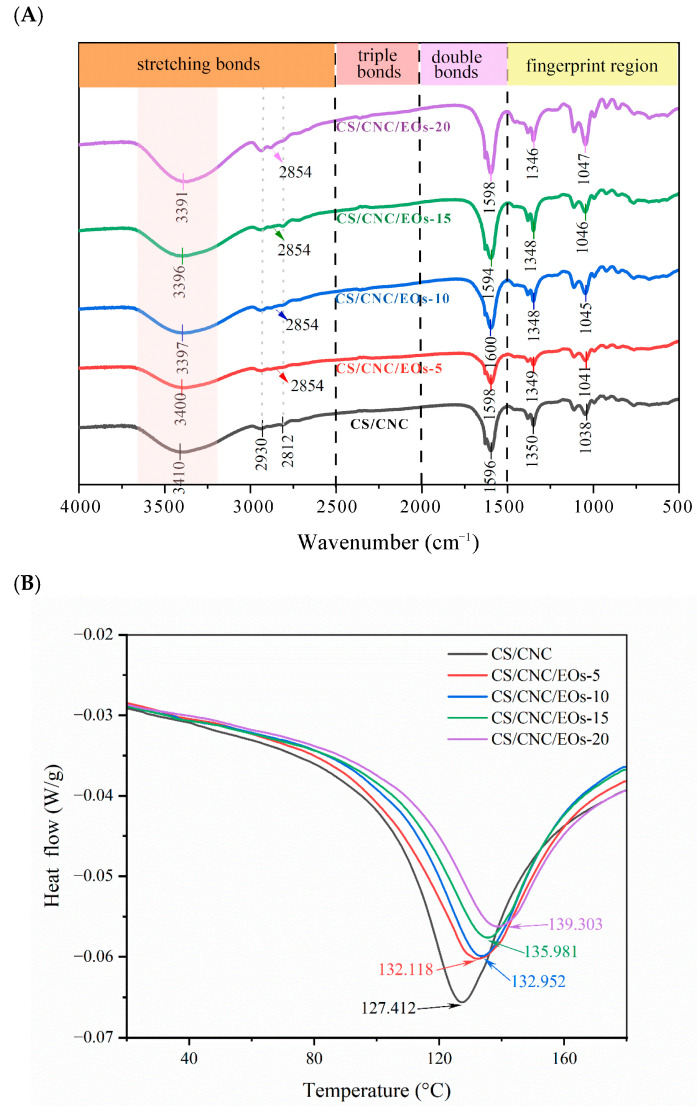
(**A**) Effect of the addition of EOs Pickering emulsions on the FTIR spectra of composite films; (**B**) DSC of nanocomposite films containing different concentrations of EOs Pickering emulsions.

FTIR was used to characterize the changes in the chemical bonds and functional groups of the packaging films at the molecular level. When major functional groups interact with each other or other functional groups, their vibrational and spectral positions change [64]. Figure 6A shows the infrared spectra of Pickering emulsion composite films containing different concentrations of EOs. The characteristic spectra of CH_3_, CH_2_, and CH tensile and bending vibrations were observed at 2930 cm^−1^ and 2812 cm^−1^. The prominent peak observed at 3413 cm^−1^ was indicative of CS and could be attributed to the simultaneous stretching vibrations of O-H and N-H in that specific spectral region. The addition of EOs Pickering emulsions not only widened the peak at 3413 cm^−1^ but also shifted this peak toward a low wave number. This indicated that hydrogen bonds were formed between the CS and the EOs Pickering emulsions [65], reducing the number of free hydroxyl groups in the active film and decreasing peak strength [37]. After the introduction of EOs Pickering emulsions, a new peak appeared near 2854 cm^−1^. This peak was attributed to the C-H tensile vibrations in EOs Pickering emulsions and became stronger as the content of the emulsion increased. The absorption peak at 1596 cm^−1^ corresponded to the -NH bending vibration. As the concentration of EOs in the Pickering emulsions increased, the absorption peak shifted toward a higher value. The change in the absorption peak was ascribed to the stretching vibration of the C=C bond in the aromatic rings present in the molecular constituents of the EOs Pickering emulsions [66]. Further, the CS neutralization process transformed the -NH^3+^ groups in the membrane into unbound amine groups [67]. As the amount of EOs added to the EOs Pickering emulsions increased, the absorption peak at 1346 cm^−1^ shifted toward the right due to C-N tensile vibrations. The decreased strength of these absorption peaks could be due to interaction (hydrogen bonding) between the -NH or -COOH groups of CS and the -OH groups of EOs Pickering emulsions. The absorption peak at 1038–1048 cm^−1^ corresponded to the ether-group C-O tensile vibrations in CS [68].

In general, after adding EOs Pickering emulsions to a thin CS film matrix, the position and intensity of the FTIR peaks were slightly altered. This demonstrated the efficient incorporation of the EOs Pickering emulsions in the CS matrix and pointed to the presence of various interactions, such as hydrogen bonds and electrostatic interaction, between CS and the EOs Pickering emulsions [69].

#### 3.5.3. Differential Scanning Calorimetry

The thermal characteristics of packaging films are crucial for preserving their long-term functionality during storage and usage [11]. Figure 6B shows the thermal properties of the films prepared in this study. In the temperature range of 100–150 °C, endothermic peaks were observed for all the film samples. An endothermic peak at about 127 °C was observed in the DSC thermogram of the CS/CNC film. This thermal event has been shown to be related to the melting transition of chitosan [70]. The incorporation of EOs Pickering emulsions in the films significantly (*p* < 0.05) influenced the endothermic peak, resulting in the shift in some heat transitions from lower to higher temperatures. As the concentration of EOs Pickering emulsions in the films increased (0.5–2.0%), the temperature at which the films were denatured increased from 132.1 °C to 139.3 °C. This could be due to the lower water content of the films resulting from the hydrophobicity of the EOs and leading to high thermal stability [71]. Interestingly, studies have shown that several of the phenolic and aldehydic constituents of EOs are extremely thermally stable [72]. In line with these reports, the films containing EOs Pickering emulsions showed a slightly higher denaturation temperature. A similar phenomenon was previously observed in CS–gelatin films after the addition of Pickering emulsions containing CIN [39]. These results confirmed that higher concentrations of EOs Pickering emulsions increase the thermal resistance of CS-based packaging films. In addition, CS/CNC/EOs films have been shown to be thermally stable at up to 90 °C, making them suitable for use in the food industry. The sausage storage experiments detailed below also confirmed the promising use of films in food packaging, especially for meat products.

### 3.6. Analysis of the Fresh-Keeping Effect of Composite Membranes

#### 3.6.1. pH

**Figure 7:**
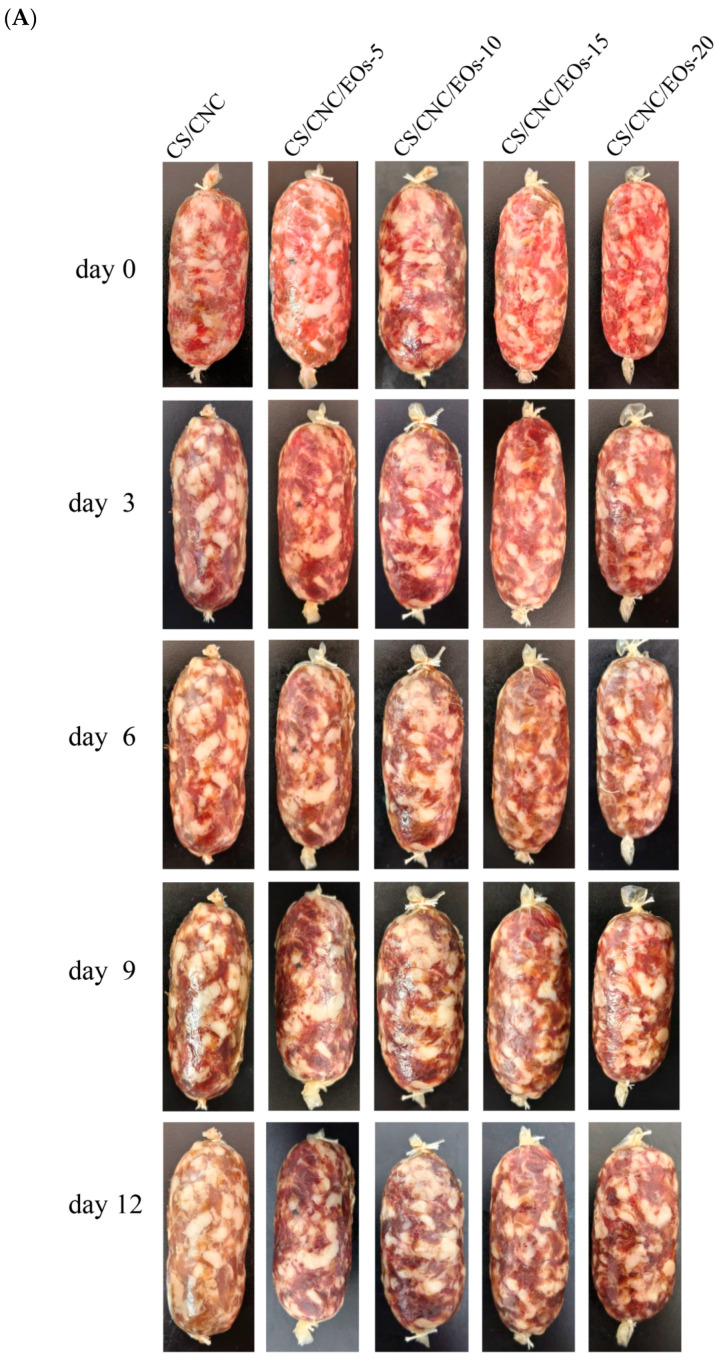

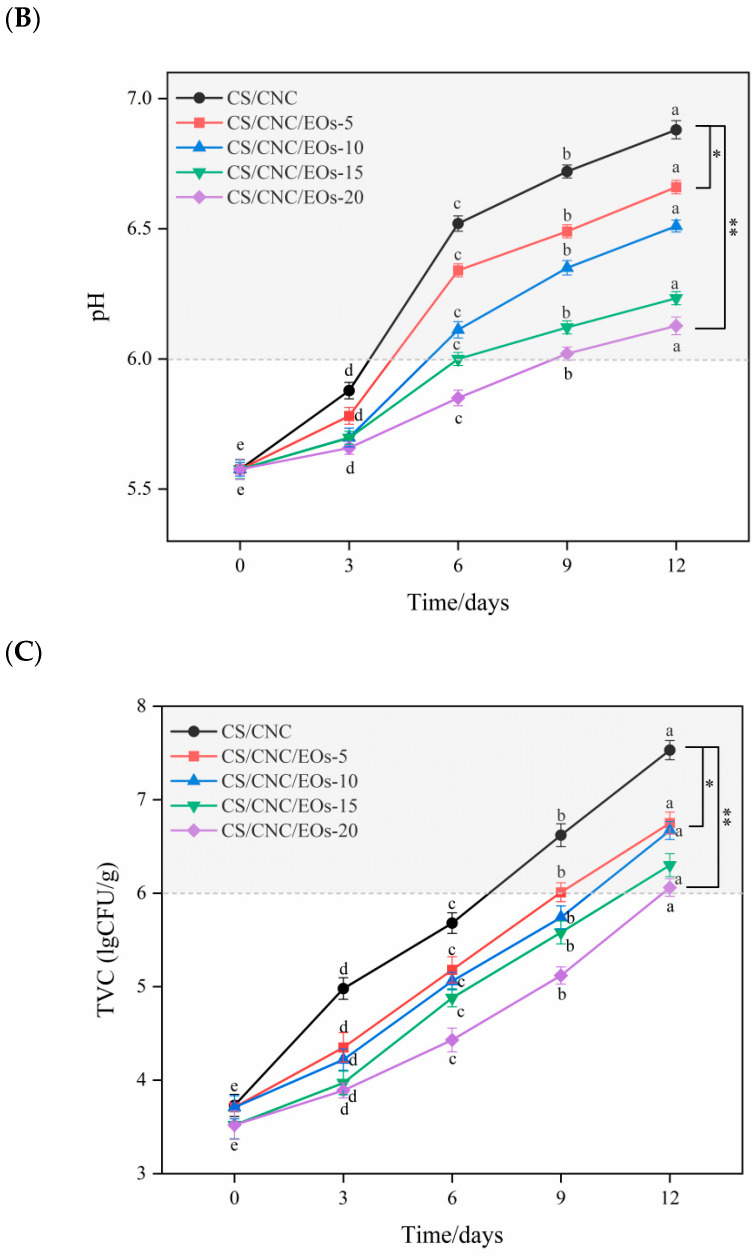

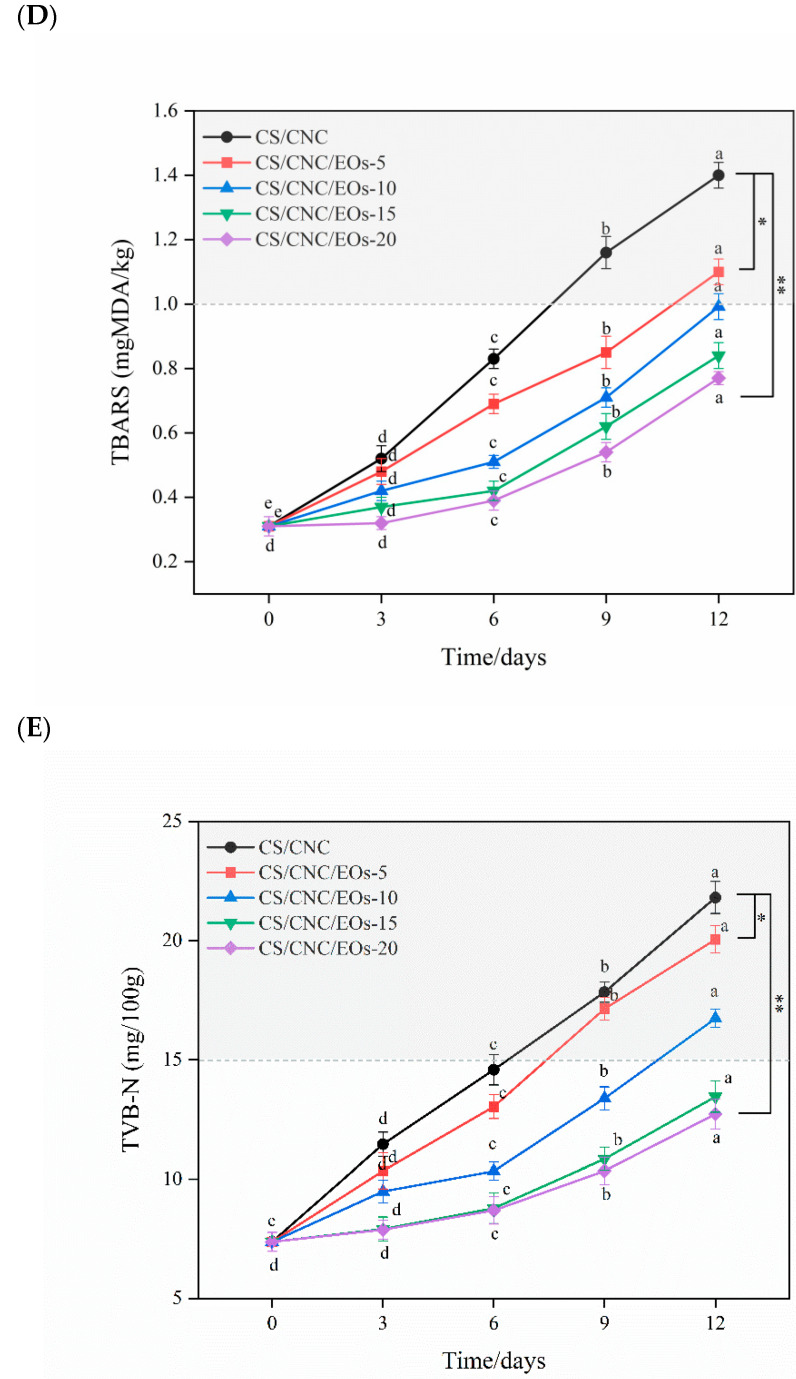
(**A**) Visual appearance of sausages packaged using different CS/CNC/EOs films during storage at 4 °C. The effect of CS/CNC/EOs films on quality parameters—such as (**B**) pH, (**C**) TVC, (**D**) TBARS, and (**E**) TVB-N—during storage. Different letters, * (*p* < 0.05)and ** (*p* < 0.01) indicate significant differences.

pH is a crucial determinant of meat product quality and directly impacts the stability of meat proteins. During the storage period, the pH of the sausages tended to increase (Figure 7B). This was likely due to the alkaline by-products generated during microbial growth, as well as the deamination of proteins [38]. However, the increase in pH was significantly slower in the CS/CNC/EOs film groups than in the control group (*p <* 0.05). Notably, with the increase in EOs concentrations, the pH elevations slowed down. This could be attributed to the antioxidant and antibacterial properties of the EOs. Several studies have demonstrated that cinnamaldehyde and its derivatives inhibit microbial growth by downregulating ATPase activity, impeding cell wall biosynthesis, and altering cell membrane structure and integrity [73]. In this study, the pH in the CS/CNC/EOs-20 group was 6.13 after 12 days of storage, which is significantly lower than that in the other groups. This indicated that composite membranes with EOs concentrations of 1.5% and 2% were the most effective at delaying protein oxidation and microbial growth, thereby inhibiting pH elevations and extending the shelf life of the packaged sausages.

#### 3.6.2. Total Viable Count

The TVC values of the sausage samples are shown in Figure 7C. The TVC in each group increased as the storage duration was prolonged, but the rate of increase in the EOs Pickering emulsion groups was lower than that in the CS/CNC group. The TVC at the start of storage was 3.52 log CFU/g. After 6 days of storage, all samples had a TVC below the recommended maximum of 6 log CFU/g. However, the TVC of sausages packed in EOs Pickering emulsion composite membranes was significantly lower than that of sausages packed in control CS/CNC membranes. After 12 days of storage, only the CS/CNC/EOs-15 and CS/CNC/EOs-20 groups showed acceptable TVC values. This could be due to the higher concentrations of EOs in these membranes. Notably, the phenol and aldehyde compounds present in EOs are known to reduce host–ligand adhesion, control bacterial metabolism, and neutralize bacterial toxins [74]. EOs can suppress the production of cellular energy, hinder the intake and utilization of glucose, and disrupt cell membrane permeability [61,75], thus inhibiting microbial growth.

#### 3.6.3. Thiobarbituric Acid Reactive Substances

Lipid oxidation can occur during the storage of meat products. A TBARS value of 1.0 mg of MDA/kg signifies that the meat is of unacceptable quality [76]. As shown in Figure 7D, the TBARS value in the CS/CNC group was less than 0.50 mg of MDA/kg after 3 days of storage, because CS itself demonstrated some antioxidant effects. Xin et al. [76] reported similar findings in their study on selenium-rich chicken sausages containing CS nanoemulsions. They observed that the CS monomer, which contains one amino group and two hydroxyl groups, can interact with free radicals and exhibit antioxidant effects. In addition, in the present study, we found that sausages wrapped in CS/CNC/EOs-20 membranes had the lowest TBARS values on day 12 (0.77 mg MDA/kg). In general, the low TBARS values for these samples could be attributed to the presence of eugenol and cinnamaldehyde, which contain a large number of unsaturated double bonds and can scour free radicals. Notably, CL can capture free radicals due to the hydrogen-donating capacity of eugenol. This prevents the formation of hydroxyperoxides and potentially inhibits the production of secondary oxidation products [77]. Meanwhile, the capacity of CIN to inhibit lipid oxidation is derived from its phenolic constituents, which suppress reactive oxygen species levels. Consequently, the films that contained EOs Pickering emulsions exhibited potent antioxidant properties and significantly prolonged the storage duration of packaged food items. The appearances of the sausages corroborate these findings, as shown in Figure 7A.

#### 3.6.4. Total Volatile Basic Nitrogen (TVB-N)

TVB-N is a crucial marker of the oxidative breakdown of proteins and amines. It can thus be used to evaluate the freshness of meat and other food products. The changes in the TVB-N values of the sausage samples prepared in this study are shown in Figure 7E. During the 12-day storage period, the TVB-N values of all the samples increased significantly (*p* < 0.05) due to microbial growth and the endogenous protease activity of meat, leading to protein degradation. However, the TVB-N values of the samples stored in EO-containing films were significantly lower than those of control samples (*p* < 0.05). In the CS/CNC group, the initial TVB-N content was 7.37 mg/100 g, but this value increased to 21.81 mg/100 g after 12 days. Similar findings were reported by Azarifar et al. [78]. Overall, the treated samples exhibited lower TVB-N values owing to decreased bacterial growth. This was due to the antimicrobial properties of CS and the antibacterial activity of the EOs Pickering emulsions incorporated into the packaging membranes. Additionally, the decreased oxidative deamidation of non-protein nitrogen compounds may have also contributed to this effect [79]. Protein oxidation can be triggered by the binding of secondary lipid oxidation products to active side chain groups. Therefore, the films containing EOs Pickering emulsions could effectively delay protein oxidation and offered significant benefits in extending the shelf life of packaged sausages.

#### 3.6.5. Sensory Analysis

**Figure 8:**
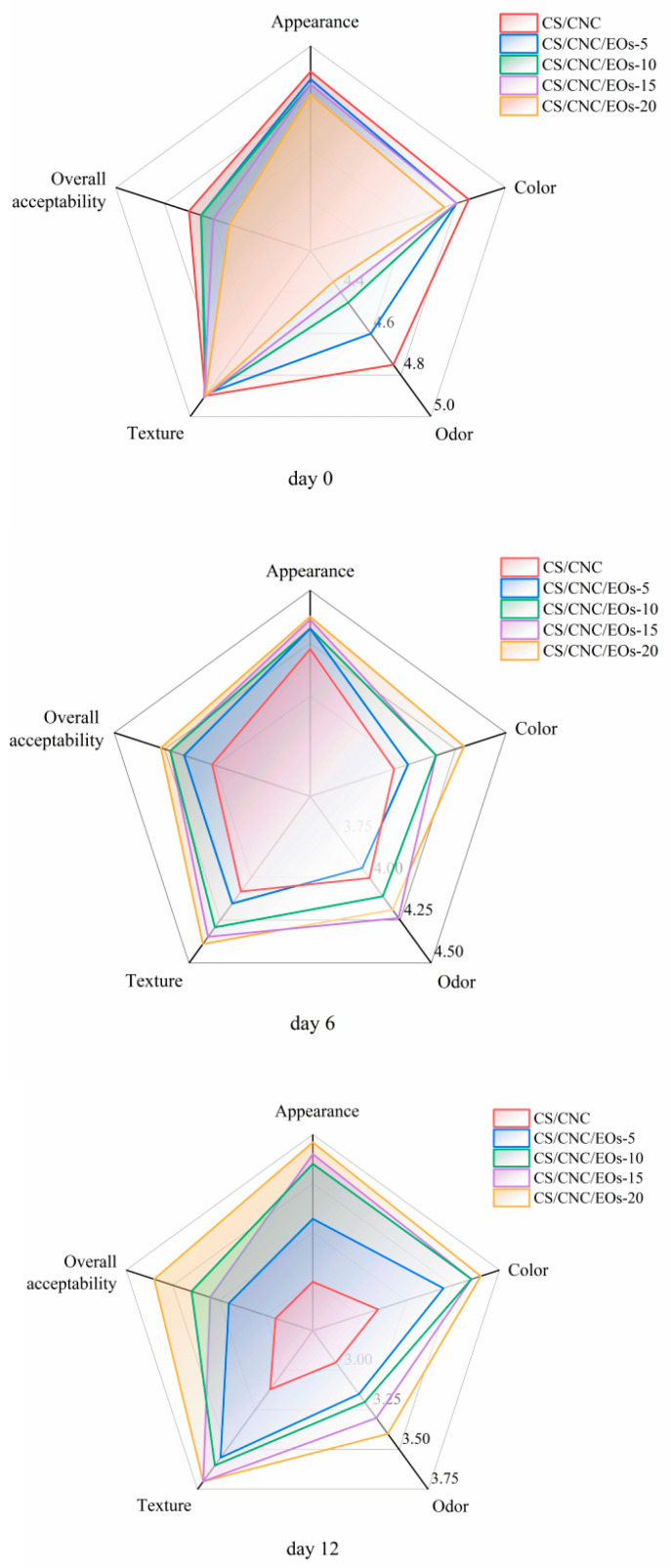
Sensory characteristics of sausages packed in films containing different concentrations of EOs during 12 days of storage at 4 °C.

As shown in Figure 8, sausages packed in films containing different concentrations of EOs showed variations in sensory properties during storage. The sensory scores gradually decreased as the storage time increased. However, the odor and color changes were slower in the EOs Pickering emulsion groups because the antioxidant and antimicrobial substances in the EOs inhibited lipid oxidation and microbial spoilage. On day 0, the panelists gave the Pickering emulsion samples supplemented with EOs a lower rating than the CS/CNC samples. This could be due to the unique flavor conferred by CIN. However, as the storage time increased, the EOs Pickering emulsion film groups scored higher than the CS/CNC group. This may be because the flavor of the EOs mellowed over time and was more palatable than that of spoiled sausages. Overall, the sensory scores of all samples decreased as the storage time increased. At 12 days, the CS/CNC group scored less than 3, indicating that the samples were not acceptable, while the EOs Pickering emulsion group samples remained acceptable. Hence, the EOs Pickering emulsion films extended the shelf life of the sausage samples, with the CS/CNC/EOs-20 film providing the best results. These findings are in line with the results of our microbial and physicochemical analysis. Similarly, Tsitsos et al. [80] reported that CS packaging films coated with oregano EOs could significantly improve the sensory properties of mutton.

#### 3.6.6. Application in Sausage Preservation

As shown in Figure 7A, the ability of the films to retain the freshness of foods was evaluated using sausage samples. The sausages were stored at 4 °C for 12 days. Initially, on day 0, all the sausages were the same color, indicating that the films containing EOs had no adverse effect on their color. Notably, the sausages showed varying degrees of deterioration by day 9. However, the sausages treated with the CS/CNC/EOs-20 film showed the slowest rate of deterioration. On day 12, the extent of color deterioration was significantly less in the sausages treated with the CS/CNC/EOs film than in those treated with the CS/CNC film. This can be attributed to the antimicrobial effect of the EOs in the film, which inhibited microbial growth, and their antioxidant capacity, which effectively slowed down the spoilage of the sausages.

Hence, the CS/CNC/EOs film, a green bio-composite packaging material, improved the preservation of sausages. Thus, it can be widely applied for the packaging of meat products.

## 4. Conclusions

In this study, we successfully developed a series of fully bio-based, antimicrobial, and antioxidant CNC/CS composite films loaded with EOs by incorporating CNC-stabilized Pickering emulsions of EOs into a CS film matrix. The composite EOs mixture containing a CIN:CL volume ratio of 2:1 had the best antimicrobial and antioxidant effects, and the Pickering emulsions of the EOs had an average particle size of 8.91 μm as well as excellent physical stability. The presence of EOs in the Pickering emulsions greatly increased the thickness of the films, reduced their WVP and EAB, and enhanced their thermal stability. During the 12 days of storage, the composite films containing EOs Pickering emulsions could slow down the increase in the pH and TVB-N values of the sausages, effectively inhibiting color changes in the meat samples, the oxidation of beef fat, and microbial growth and reproduction. Accordingly, the composite films containing EOs Pickering emulsions prolonged the shelf-life of the sausages. This study provides a reliable theoretical basis for the large-scale industrial application of these films as natural freshness-preserving packaging materials for sausages and other products. The findings demonstrate the potential for EOs Pickering emulsion composite films prepared using CS to be as the matrix material for active food packaging.

## Figures and Tables

**Table 1:** Minimum inhibitory concentration (MIC) and inhibitory concentration index (FICI) values of individual and composite essential oils against different bacterial and fungal strains.

Microorganisms	MIC (μg/mL)				FIC (CIN)	FIC (CL)	FICI	Remarks
	CIN	CL	EOs (CIN)	EOs (CL)				
*Bacillus cereus* (ATCC 14579)	0.5	1.0	0.125	0.25	0.25	0.25	0.5	S
*Salmonella enterica* (ATCC 12002)	0.25	1.0	0.125	0.125	0.5	0.125	0.625	ADD
*Staphylococcus aureus* (ATCC 6538)	0.25	0.5	0.0313	0.125	0.125	0.25	0.375	S
*Escherichia coli* (ATCC 8739)	0.25	1.0	0.125	0.5	0.5	0.5	1.0	ADD
*Aspergillus niger* (ATCC 16404)	0.125	0.5	0.0625	0.25	0.5	0.5	1.0	ADD
*Pseudomonas aeruginosa* (ATCC 9027)	1.0	2	0.5	0.5	0.5	0.25	0.75	ADD

CIN: cinnamon essential oil; CL: clove essential oil; EOs (*): MIC values of * essential oil combinations. S: synergism (FICI ≤ 0.5); ADD: additive (0.5 < FICI ≤ 1.0); INN: indifferent (1.0 < FICI ≤ 4.9); ANT: antagonism (FICI > 4.0).

**Table 2:** Thickness, tensile strength (TS), elongation at break (EAB), and water vapor permeability (WVP) of the CS/CNC/EOs films.

Film Samples	Thickness	TS	EAB	WVP × 1010
	mm	MPa	%	g·m^−1^s^−1^Pa^−1^
CS/CNC	0.114 ± 0.007 ^a^	19.43 ± 0.57 ^a^	24.33 ± 0.62 ^a^	4.38 ± 0.17 ^a^
CS/CNC/EOs-5	0.126 ± 0.009 ^b^	13.98 ± 0.64 ^d^	17.55 ± 0.64 ^d^	3.65 ± 0.21 ^b^
CS/CNC/EOs-10	0.134 ± 0.009 ^c^	14.17 ± 0.56 ^d^	19.39 ± 0.83 ^c^	3.56 ± 0.18 ^b^
CS/CNC/EOs-15	0.153 ± 0.011 ^d^	16.89 ± 0.62 ^b^	20.74 ± 0.59 ^b^	2.93 ± 0.13 ^c^
CS/CNC/EOs-20	0.161 ± 0.014 ^e^	15.52 ± 0.59 ^c^	18.98 ± 0.73 ^c^	2.14 ± 0.15 ^d^

Values are presented as the means ± standard deviations (*n* = 3). Different superscripts in the same column indicate significant differences between different films (*p* < 0.05).

**Table 3:** Colors of films containing different concentrations of Pickering emulsions.

Film Samples	L*	a*	b*	ΔE
CS/CNC	93.02 ± 0.31 ^a^	−1.46 ± 0.27 ^c^	2.45 ± 0.09 ^e^	5.41 ± 0.21 ^e^
CS/CNC/EOs-5	91.94 ± 0.42 ^b^	−1.31 ± 0.28 ^c^	9.78 ± 0.40 ^d^	11.21 ± 0.37 ^d^
CS/CNC/EOs-10	89.17 ± 0.33 ^c^	−0.88 ± 0.31 ^b^	11.62 ± 0.38 ^c^	14.26 ± 0.34 ^c^
CS/CNC/EOs-15	87.45 ± 0.53 ^d^	1.05 ± 0.21 ^a^	12.89 ± 0.51 ^b^	16.34 ± 0.45 ^b^
CS/CNC/EOs-20	87.06 ± 0.45 ^d^	1.23 ± 0.18 ^a^	14.51 ± 0.64 ^a^	17.89 ± 0.48 ^a^

Values are presented as the means ± standard deviations (n = 3). Different superscripts in the same column indicate significant differences between different films (*p* < 0.05).

## Data Availability

The original contributions presented in the study are included in the article, further inquiries can be directed to the corresponding author.
